# Development and validation of an osteoporosis risk prediction model incorporating nighttime eating exposure

**DOI:** 10.3389/fnut.2025.1660080

**Published:** 2025-11-21

**Authors:** Chenxi Liu, Yiping Tong, Mingxia Pan, Yang Li, Xuan Yang, Yanlei Wang, Lingbo Xing

**Affiliations:** 1 Luoyang Orthopedic Hospital of Henan Province (Henan Provincial Orthopedic Hospital), Luoyang, China; 2 School of Nursing, Fujian University of Traditional Chinese Medicine, Fuzhou, China

**Keywords:** chrono-nutrition, nighttime eating exposure, osteoporosis, Mendelian randomization, bone mineral density

## Abstract

**Introduction:**

Osteoporosis (OP) is a global bone metabolic disease, and its incidence continues to rise with the intensifying aging of the population. In recent years, chrono-nutrition research has uncovered the significant impact of dietary timing patterns on health, but the relationship between nighttime eating exposure (NEE) and OP remains unclear.This study aimed to confirm through multi-dimensional methodologies that NEE is an independent risk factor for OP.

**Methods:**

A clinical cross-sectional study (*n* = 186) employed LASSO regression, logistic regression, and random forest to screen for OP predictors, constructing and validating a nomogram model. Analysis of the US NHANES database (*n* = 18,975) assessed the association between NEE and OP and explored non-linear relationships using weighted logistic regression. Multivariable Mendelian randomization (MR) analysis inferred causal associations using genetic instruments for dietary patterns, BMI, and sleep duration.

**Results:**

NEE was identified as an independent risk factor for OP. In the clinical prediction model, the combination model including NEE (NEE + bone mineral density + age, etc.) demonstrated optimal predictive performance (training set AUC = 0.808). NHANES validation showed a significantly increased OP risk when NEE > 25% (OR = 1.83, 95% CI: 1.27–2.64), with a dose-effect threshold identified (steep risk increase at NEE = 10–25%). Subgroup analysis revealed that the effect size of NEE was significantly higher in individuals with osteopenia (OR = 1.12) compared to those with normal bone mass (OR = 1.06, interaction *p* = 0.0297), and the protective effect of bone mineral density was weakened in the osteopenic group. Multivariable MR results indicated that a genetic predisposition representing dietary disturbance was positively associated with OP risk (*β* = 0.0228, *p* = 0.0033), reflecting the potential causal role of NEE-related behavioral patterns.

**Discussion:**

NEE is a novel behavioral risk factor for OP, posing a significant hazard particularly for osteopenic individuals. Its mechanism may be related to circadian rhythm disruption, metabolic dysregulation, and melatonin suppression. This research provides a new direction for precision OP prevention strategies based on “chrono-nutrition.”

## 1 Introduction

Osteoporosis (OP) is a systemic metabolic disease primarily caused by an imbalance in bone metabolism (1). With the intensification of global population aging, its incidence continues to rise, and it is projected to affect over 200 million people worldwide (2). Therefore, in-depth exploration of effective early prevention and intervention strategies for OP holds significant importance for research in this field.

Chrono-nutrition, an emerging field, investigates the impact of the circadian rhythm of dietary behaviors on health, emphasizing the importance of the timing of food intake beyond merely its quantity and quality (3). Nutritional imbalances can remodel the circadian clock, and food consumption at different times has been demonstrated to profoundly affect physiological functions (4, 5). Accumulating evidence indicates that disruptions in circadian rhythms and dietary patterns, such as skipping breakfast, consuming high-energy foods at dinner, and late-night eating, are associated with various health hazards Unlike traditional research focusing on “what” and “how much” to eat, this study explores the value of specific dietary timing behaviors on skeletal health.

Nighttime Eating Exposure (NEE) refers to the consumption of food, particularly energy-dense items such as high-fat and high-sugar processed foods, during the biological rest period, which typically occurs at night. As prior research has not explicitly defined the temporal boundaries for nighttime eating exposure, based on a review of the literature and consideration of lifestyle habits and circadian rhythms, this study provisionally defines the NEE window as 8:00 p.m. to 6:00 a.m. (6, 7). This dietary behavior may pose a potential threat to skeletal health and exacerbate OP risk. It is hypothesized that this eating pattern may disrupt bone metabolic homeostasis through several pathways: First, it may directly disrupt the rhythmic expression of core circadian clock genes, suppress osteoblast activity, and potentially enhance osteoclast function, disrupting the balance between bone formation and resorption (8). Concurrently, it may lead to skeletal muscle mitochondrial dysfunction; for example, BMAL1 and CLOCK knockout mice exhibit reduced mitochondrial volume in muscle,morphological defects in remaining mitochondria, decreased respiratory function, and reduced PGC-1α levels, resulting in diminished muscle contractility (9, 10). Second, nighttime eating is often accompanied by metabolic disturbances, such as decreased insulin sensitivity (11), impaired glucose tolerance, and adipose tissue dysfunction. These metabolic alterations may indirectly lead to the upregulation of inflammatory factors or affect bone metabolism-related hormones, such as leptin and adiponectin, thereby negatively regulating bone remodeling processes (12). Moreover, consuming food at night exposes individuals to artificial light, which may inhibit the secretion of melatonin. Research has demonstrated that melatonin possesses a direct bone-protective effect. (13); thus, chronic nighttime light exposure may further impair bone mineralization capacity. Given these considerations, chronic irregular nighttime eating habits constitute an important chrono-nutritional risk factor. By interfering with endogenous circadian rhythms, inducing metabolic dysregulation, and affecting key hormones, NEE may collectively contribute to reduced bone mass and degradation of bone microstructure, ultimately promoting the onset and progression of OP.

**Figure 1:**
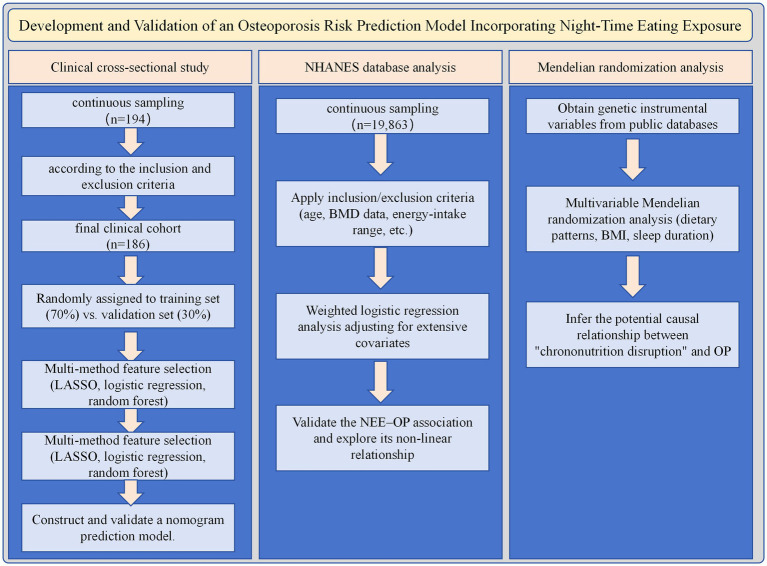
Flowchart of the study design.

Chrono-nutrition research has confirmed significant associations between dinner intake patterns and health outcomes, but the specific impact of NEE on OP and its underlying mechanisms remain unclear. To preliminarily investigate the association between NEE and OP, this study employed a three-part methodology: first, conducting a clinical cross-sectional analysis to construct an NEE-OP risk prediction model; second, utilizing the US National Health and Nutrition Examination Survey (NHANES) database to validate this association in a large, nationally representative sample and explore dose–response relationships (14); finally, applying Mendelian Randomization (MR) analysis to infer potential causal relationships while controlling for confounding factors and reverse causality (15). This research strategy, which advances from local clinical samples to nationwide large-sample validation and subsequently to genetic causal inference, aims to establish a robust, step-by-step, and comprehensive foundation for transitioning from association to causation regarding the novel risk factor of ‘NEE’.(See Figure 1).

## 2 Materials and methods

### 2.1 Study design and participants

This cross-sectional study aimed to investigate the association between nighttime eating exposure (NEE, exposure) and osteoporosis (outcome). The sample size was calculated using the formula for cross-sectional surveys (*α* = 0.05, Z₂/*α* = 1.96). As shown in Equation (1).


(1) $$n=\frac{{Z^{2}}_{a/2}(1-p)p}{\delta^{2}}$$

Based on an osteoporosis patient prevalence *p* = 39.19% (16), and an allowable error *δ* = 0.2p = 0.07838, the minimum sample size was calculated as 150. The final sample size was determined to be 150 participants. This study employed consecutive sampling from patients with nighttime eating habits at the Neck, Shoulder, Low Back, and Leg Pain Center of Luoyang Orthopedic Hospital (Henan Provincial Orthopedic Hospital) from January 2024 to May 2025. Inclusion criteria: (1) Patients with nighttime eating habits; (2) Able to undergo both lumbar spine and femoral neck bone mineral density examinations within an interval not exceeding 7 days to minimize external influences and ensure consistency; (3) Age 18 years or older; clear consciousness, possessing normal reading and communication abilities. Exclusion criteria: (1) Presence of congenital scoliosis, spinal fracture, spinal tumor, ankylosing spondylitis; (2) Suffering from diseases severely affecting bone metabolism, such as chronic kidney/liver failure, hyperparathyroidism, rheumatoid arthritis, and other autoimmune diseases; (3) Long-term use of medications affecting bone metabolism, such as glucocorticoids, antiepileptic drugs, etc.; (4) Patients with incomplete questionnaires or clinical data. Osteoporosis diagnosis in enrolled patients followed the Guidelines for Diagnosis and Treatment of Primary Osteoporosis (2022) (17). Informed consent was obtained from all participants prior to any procedures. Before any procedure, all participants provided written informed consent. The study was conducted in accordance with the principles and guidelines of the Declaration of Helsinki and was approved by the Ethics Committee of Henan Luoyang Orthopedic Hospital (Henan Orthopedic Hospital) (approval No. 2023KYKT0021-02).

### 2.2 Nighttime eating exposure

During the dietary assessment, rigorously trained researchers employed the Retrospective Dietary Survey Auxiliary Reference Food Atlas, developed by Professor Wang Zhixu, alongside food models to assist patients, family members, or caregivers in recalling dietary intake over a period of three days. The average daily nutrient intake was calculated using Nutrition Calculator v2.8.0.5, provided by the Institute of Nutrition and Food Safety, Chinese Center for Disease Control and Prevention, Beijing, China. This study utilized a 24-h dietary recall form, where dietary intake was evaluated through face-to-face interviews conducted over three consecutive days. All patients exhibiting nighttime eating habits were interviewed to describe the types and quantities of foods consumed within the past 24 h. In cases where patient recall proved challenging, family members supplemented the dietary information. All dietary surveys were conducted following patient admission. The timing of food intake was incorporated into the standard 24-h dietary recall form. Due to the absence of a standardized definition in prior research, the NEE window was provisionally defined as occurring between 8:00 p.m. and 6:00 a.m., based on relevant literature and dietary habits. It is important to note that this study is a preliminary exploration of the relationship between NEE and obesity prevalence (OP); therefore, special circumstances, such as night-shift workers, were not considered separately, and only food consumption within this defined time window was included. The calculation formula for NEE is as shown in Equation (2):


(2) $$\mathrm{NEE}=\frac{\text{night}\_\text{kcal}}{\text{total}\_\text{kcal}}\times 100\%$$

### 2.3 Data collection and quality control

Investigators personally distributed questionnaires and provided detailed explanations to participants regarding the survey’s purpose, importance, requirements, and completion methods. All questionnaires were collected immediately upon completion by patients and meticulously checked by investigators for completeness and accuracy. A total of 194 questionnaires were distributed, with 186 valid questionnaires ultimately collected, yielding an effective response rate of 95.6%. Patient disease data were collected and entered by investigators via the hospital’s nursing information system, with each entry verified individually.

### 2.4 Predictive model construction and validation

Data Preparation and Splitting: The osteoporosis status was converted into a binary factor variable (positive/negative). A stratified random sampling strategy (strata = “osteoporosis”) was employed to split the dataset into training (70%) and testing (30%) sets at a 7:3 ratio. Reproducibility was ensured using set.seed(123). Stratified sampling guaranteed consistent outcome variable distribution between groups.

Feature Engineering: Predictor variables in the training set were converted to a design matrix via the model.matrix function, with the intercept term removed ([, −1]). All continuous variables were centered and scaled, and zero-variance features (zv) were eliminated.

Feature Selection and Model Construction: A multi-method fusion strategy was used for feature selection to enhance robustness. Model construction: LASSO Regression: The glmnet package was used to fit a LASSO-regularized logistic regression model (family = “binomial,” alpha = 1). The *λ* value was optimized via 10-fold cross-validation. This step was limited to hyperparameter tuning for the LASSO model, aiming to prevent overfitting. Logistic Regression: A standard logistic regression model was fitted using identical preprocessing and 10-fold cross-validation. Random Forest: The randomForest package was used to fit an ensemble model with 500 decision trees (ntree = 500). Predictor importance was calculated based on the mean decrease in Gini impurity. The top 5 important variables from each of the three methods were selected, and their union was taken,finally incorporating 7 variables to construct the final nomogram prediction model.

Model validation: A multi-level validation strategy was employed to assess model performance: the model’s performance was evaluated on an independent test set, the Receiver Operating Characteristic curve was calculated to assess discriminative ability, and Decision Curve Analysis and Clinical Impact Curve were used to evaluate clinical utility; additionally, 1,000 Bootstrap resamplings were performed to calculate the model’s calibration C-index and Optimism, quantifying the assessment of the model’s stability and internal validity.

### 2.5 Validation of the NHANES database

We selected the NHANES database cycles 2013–2014 and 2017–2018; data were merged initially, but the resulting dataset size was small after direct processing, necessitating the use of random forest imputation for handling missing data. Based on literature review and the focus of this study investigating the relationship between NEE and OP (18), with selection results shown in Supplementary Figure 1. Dietary data were obtained using two non-consecutive 24-h dietary recall interviews following USDA Food and Nutrition Database guidelines. The first interview was conducted in-person, and the second was administered via telephone 3 to 10 days later. Dietary intake data estimated the types and quantities of all foods and beverages (including all types of water) consumed during the 24-h period (midnight to midnight) preceding each interview. Total nutrient intake profiles were generated for each participant, representing daily total energy and nutrient intake from foods and beverages. Time quantification variables were processed using the lubridate package to convert eating time strings (DR1_020) into POSIXct time objects, extracting hour and minute numerical variables. Energy intake statistics were calculated by grouping by subject ID (SEQN): total_kcal represented total daily energy intake; night_kcal represented the sum of energy intake during the nighttime period (night = 1). Data quality control measures included: excluding extreme energy intake (retaining only records with 500 ≤ total_kcal ≤ 5,000 kcal/day); handling zero values (when total_kcal = 0, night_ratio was forced to NA); and coding missing time data (original code 99) as NA. Covariates considered included age, race/ethnicity, education level, household income, marital status, smoking status, alcohol consumption, body mass index (BMI), diabetes status, hypertension status, lumbar spine bone mineral density (BMD), femoral neck BMD, and white blood cell count. The outcome variable was defined as self-reported physician-diagnosed osteoporosis/bone fragility.

### 2.6 Multi-sample Mendel

NEE served as the exposure variable in this study. As prior research lacks directly relevant exposure variables, no direct genetic instrument for NEE was identified; therefore, indirect exposure variables (sleep deprivation, 24-h dietary patterns, and BMI) were selected to investigate the relationship between NEE and OP using multivariable Mendelian randomization (MVMR). Genetic variants for dietary variables (ukb-b-14294) and BMI (ukb-b-19953) were obtained from European population studies within the UK Biobank; BMI data (ebi-a-GCST006686) were sourced from the European Bioinformatics Institute. Instrumental variable (IV) selection adhered to three core MR assumptions: First, single nucleotide polymorphisms (SNPs) must be strongly associated with the exposure (*p* < 5 × 10^−6^) and exhibit an F-statistic >10. Second, independent SNPs were ensured using linkage disequilibrium (LD) clumping (R^2^ < 0.001, distance = 10,000 kb). Finally, SNPs associated with the outcome or confounding variables were excluded (p < 5 × 10^−6^). The OP outcome data (ukb-b-12141) were derived from a European population study within the UK Biobank by Ben Elsworth et al., comprising 462,933 individuals of European ancestry, including 455.386 with OP. MR-PRESSO analysis detected no outlier SNPs.

### 2.7 Statistical analysis

First, LASSO regression (glmnet package), logistic regression (method = “glm”), and random forest (randomForest package) were applied to the training set data for feature selection to identify core predictors of osteoporosis. Subsequently, selected variables were integrated to construct a visual nomogram (rms:nomogram) for individualized risk quantification. Further, model discriminative ability was evaluated using receiver operating characteristic (ROC) curves (pROC package calculating AUC and 95% CI), while calibration curve analysis (rms:calibrate) supplemented by the Hosmer-Lemeshow goodness-of-fit test was performed on both training and validation sets to assess agreement between predicted risk and observed frequencies. Finally, decision curve analysis (DCA) (rmda:decision_curve) quantified the clinical net benefit across risk thresholds of 10–80%, comprehensively evaluating clinical utility. Analyses used R software (version 4.2.1). All NHANES analyses incorporated survey weights to account for the complex multi-stage probability sampling design. Categorical variables are presented as weighted frequencies (%), with group comparisons performed using weighted chi-square tests. Continuous variables are expressed as mean ± standard error (SE), and group comparisons used weighted linear regression models. Three multivariable logistic regression models assessed the association between NEE and OP: Crude model (unadjusted); Model 1 (adjusted for age, sex, race/ethnicity, education level, and marital status); Model 2 (further adjusted for smoking status, alcohol consumption, BMI, diabetes, hypertension, lumbar spine BMD, femoral neck BMD, and white blood cell count). Inverse-variance weighted (IVW) served as the primary method for Mendelian randomization (MR) analysis, with MR-Egger and weighted median as supplementary methods. MR findings are presented as odds ratios (ORs) per unit increase in the genetically predicted exposure. The MR-Egger intercept tested for horizontal pleiotropy (intercept close to 0 and *p* > 0.05 indicating absence). Heterogeneity among SNPs was assessed using Cochran’s Q test (p > 0.05 and I^2^ < 25% considered acceptable). MR reliability was evaluated via funnel plot symmetry and leave-one-out analysis. MR analyses used the “MRPRESSO” and “TwoSampleMR” R packages. *p* < 0.05 was considered statistically significant.

## 3 Results

### 3.1 General information of the participant

This study included 186 participants. The prevalence of osteoporosis in the overall cohort was 14.6%, with balanced distribution between the training and validation sets (14.5% vs. 14.8%). No statistically significant differences (*p* > 0.05) were observed between the training and validation sets regarding clinical characteristics including osteoporosis status, age, hypertension, diabetes, education level, height, BMI, etc. (Table 1).

**Table 1:** Baseline characteristics of the clinical cohort.

Characteristic	Overall *N* = 186^1^	Train *N* = 130^1^	Validation *N* = 56^1^	*P*
Osteoporosis				0.796
Yes	106 (57%)	76 (58%)	30 (54%)	
No	80 (43%)	54 (42%)	26 (46%)	
Gender				0.354
Male	99 (53%)	65 (50%)	34 (61%)	
Female	87 (47%)	65 (50%)	22 (39%)	
Age	49 (18)	49 (17)	50 (19)	0.192
Level of education				0.202
Less Than 9th Grade	7 (3.8%)	5 (3.8%)	2 (3.6%)	
9-11th Grade (Includes 12th grade with no diploma)	19 (10%)	14 (11%)	5 (8.9%)	
High school grad/GED or equivalent	45 (24%)	30 (23%)	15 (27%)	
College diploma (Associate degree)	61 (33%)	45 (35%)	16 (29%)	
College graduate or above	54 (29%)	36 (28%)	18 (32%)	
Marriage				0.985
Married	88 (47%)	65 (50%)	23 (41%)	
Widowed	5 (2.7%)	3 (2.3%)	2 (3.6%)	
Divorced	7 (3.8%)	4 (3.1%)	3 (5.4%)	
Separated	3 (1.6%)	3 (2.3%)	0 (0%)	
Never married	67 (36%)	46 (35%)	21 (38%)	
Living with partner	16 (8.6%)	9 (6.9%)	7 (13%)	
Income	2.78 (1.60)	2.83 (1.63)	2.65 (1.56)	0.599
BMI	26.8 (6.6)	27.0 (6.9)	26.2 (5.8)	0.491
Smoking				0.1
No	52 (28%)	36 (28%)	16 (29%)	
Yes	134 (72%)	94 (72%)	40 (71%)	
Drinking				
No	48 (26%)	33 (25%)	15 (27%)	0.281
Yes	138 (74%)	97 (74.8%)	41 (72.8%)	
White blood cell count	7.62 (2.05)	7.54 (2.06)	7.80 (2.03)	0.598
Femoral shaft density	−1.15 (1.30)	−1.16 (1.30)	−1.12 (1.32)	0.86
Insomnia				0.678
Yes	115 (62%)	78 (60%)	37 (66%)	
No	71 (38%)	52 (40%)	19 (34%)	
Lumbar done density	−1.50 (1.28)	−1.47 (1.30)	−1.58 (1.24)	
Hypertension				0.325
Yes	103 (55%)	74 (57%)	29 (52%)	
No	83 (45%)	56 (43%)	27 (48%)	
Diabetes				0.487
Yes	107 (58%)	77 (59%)	30 (54%)	
No	79 (42%)	53 (41%)	26 (46%)	
NEE	27 (12)	27 (12)	28 (12)	0.933

^1^n (%); Mean (SD).

### 3.2 Screening of influencing factors

**Figure 2:**
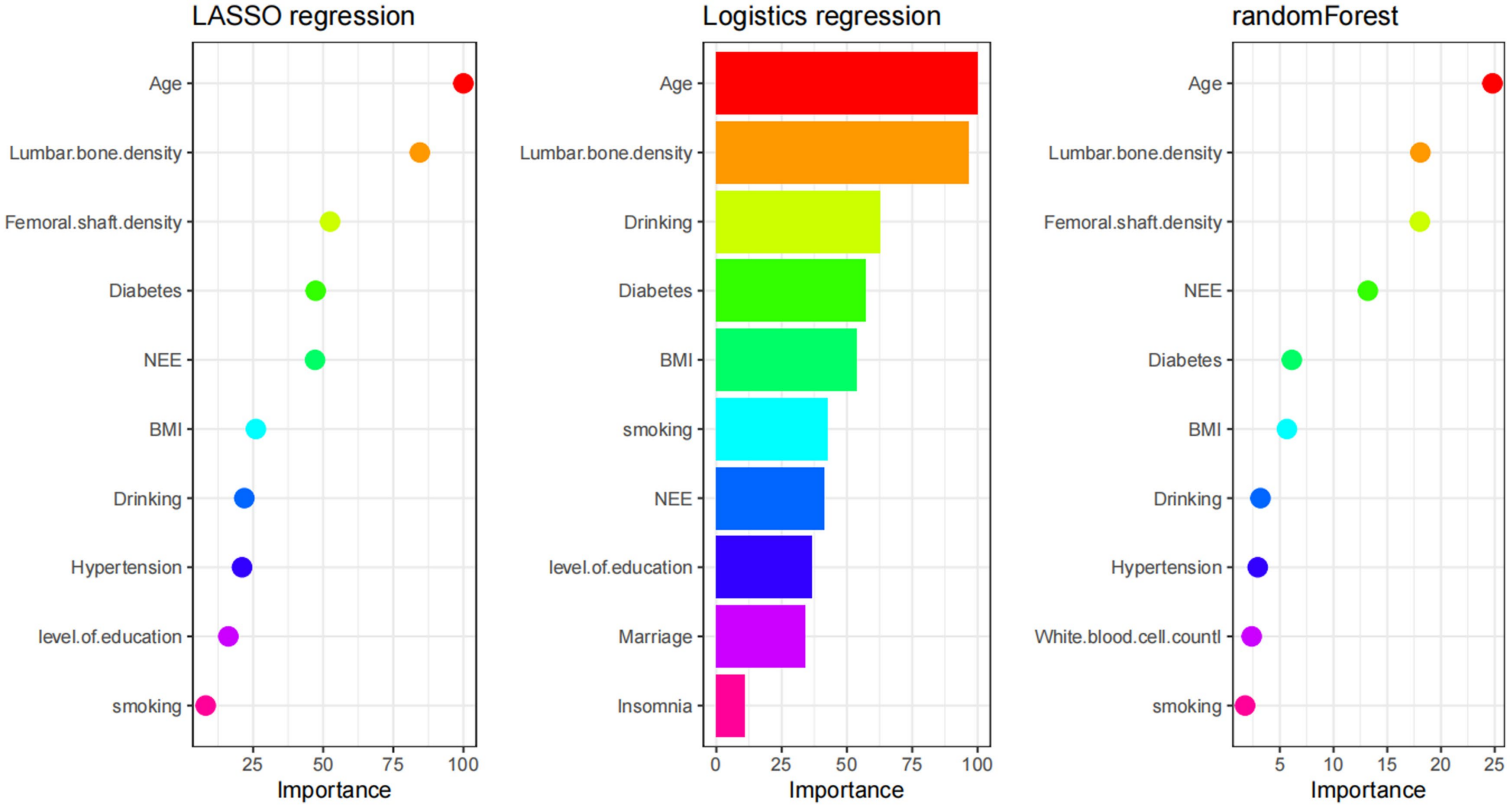
Comparison of feature importance across the three feature-selection models: the ranking of predictors for osteoporosis risk is shown for LASSO regression (left), logistic regression (middle), and random forest (right).

Analysis of factors influencing osteoporosis based on the training set, using three methods (LASSO regression, logistic regression, random forest), all identified NEE as one of the influencing factors for osteoporosis. NEE showed a moderate effect in LASSO regression and random forest, while its importance score was lower in logistic regression. From the figure, it can be seen that the importance of NEE in model validation was higher than traditional social-behavioral factors like smoking and alcohol consumption in two models, but weaker than the gold standard BMD and age, indicating its value as an independent risk factor for supplementary screening, potentially providing early warning, especially in individuals with borderline BMD values. (Figure 2).

### 3.3 Establishment of predictive models

**Figure 3:**
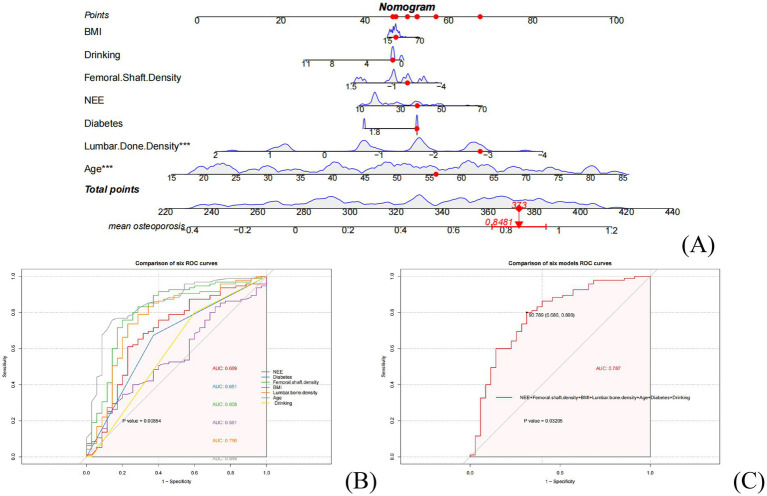
Construction and validation of the osteoporosis risk prediction model in the training set: **(A)** predictive nomogram; **(B)** Single-variable ROC curve comparisons; **(C)** Multivariable model ROC comparisons.

The influencing factors screened by the three models from the training set were used for nomogram and ROC curve analysis, constructing an osteoporosis prediction model. The nomogram model integrated key predictive variables, allowing for visual quantification of individual OP risk. Among them, NEE contributed significantly as an independent behavioral risk factor, forming a core predictive framework together with BMD indicators (lumbar/femoral). In ROC curve comparisons, the combined model including NEE (NEE + Femoral shaft density + Lumbar bone density + Age + Drinking) demonstrated high predictive performance (AUC = 0.808), significantly superior to single-variable models. This confirmed a synergistic effect between NEE and BMD indicators, and that NEE is an indispensable independent variable in the osteoporosis prediction model. (Figures 3A–C).

### 3.4 Test set validation

**Figure 4:**
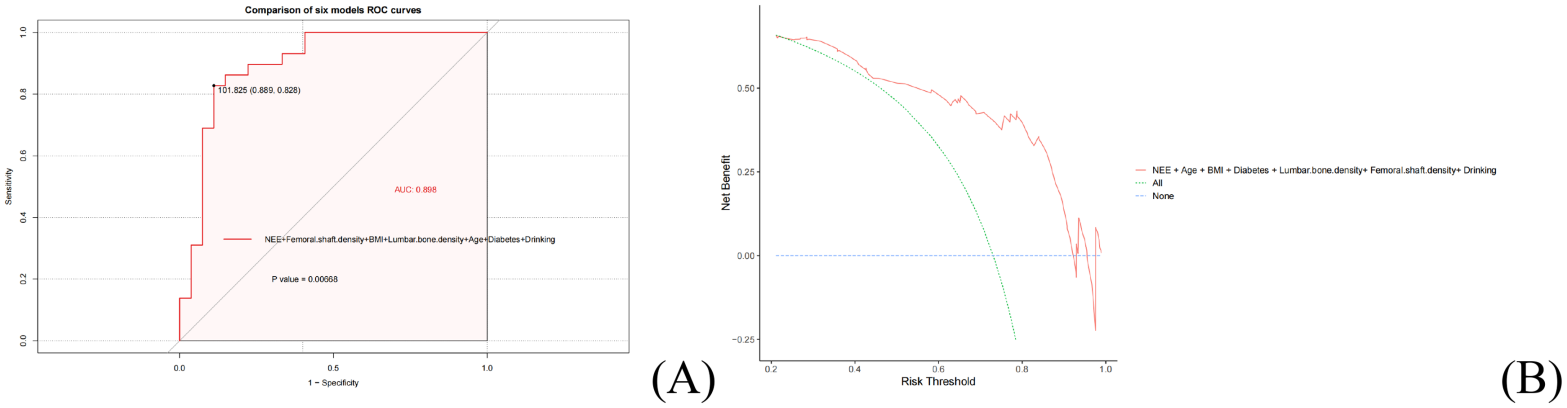
Performance evaluation of the osteoporosis prediction model in the validation set: **(A)** Multivariable ROC comparison; **(B)** Decision curve analysis (DCA).

In the validation set, this prediction model maintained good discriminative ability, with an AUC of 0.898, confirming that variable synergy enhances model discrimination. DCA further confirmed that this NEE-containing model (red curve) yielded consistently higher net benefit than the “all-variables model” (green curve) and the “null model” (blue dashed line) within the 0.1–0.6 risk threshold range, with the most substantial net benefit advantage observed specifically within the clinically critical 0.2–0.4 threshold interval (Figures 4A,B). To evaluate the stability of the nomogram’s predictive performance, Bootstrap resampling (1,000 times) showed the model’s corrected C-index was 0.795 (95% CI: 0.752–0.838), indicating low model optimism and good internal stability (Supplementary Table 1).

### 3.5 Subgroup analysis of osteopenia

**Figure 5:**
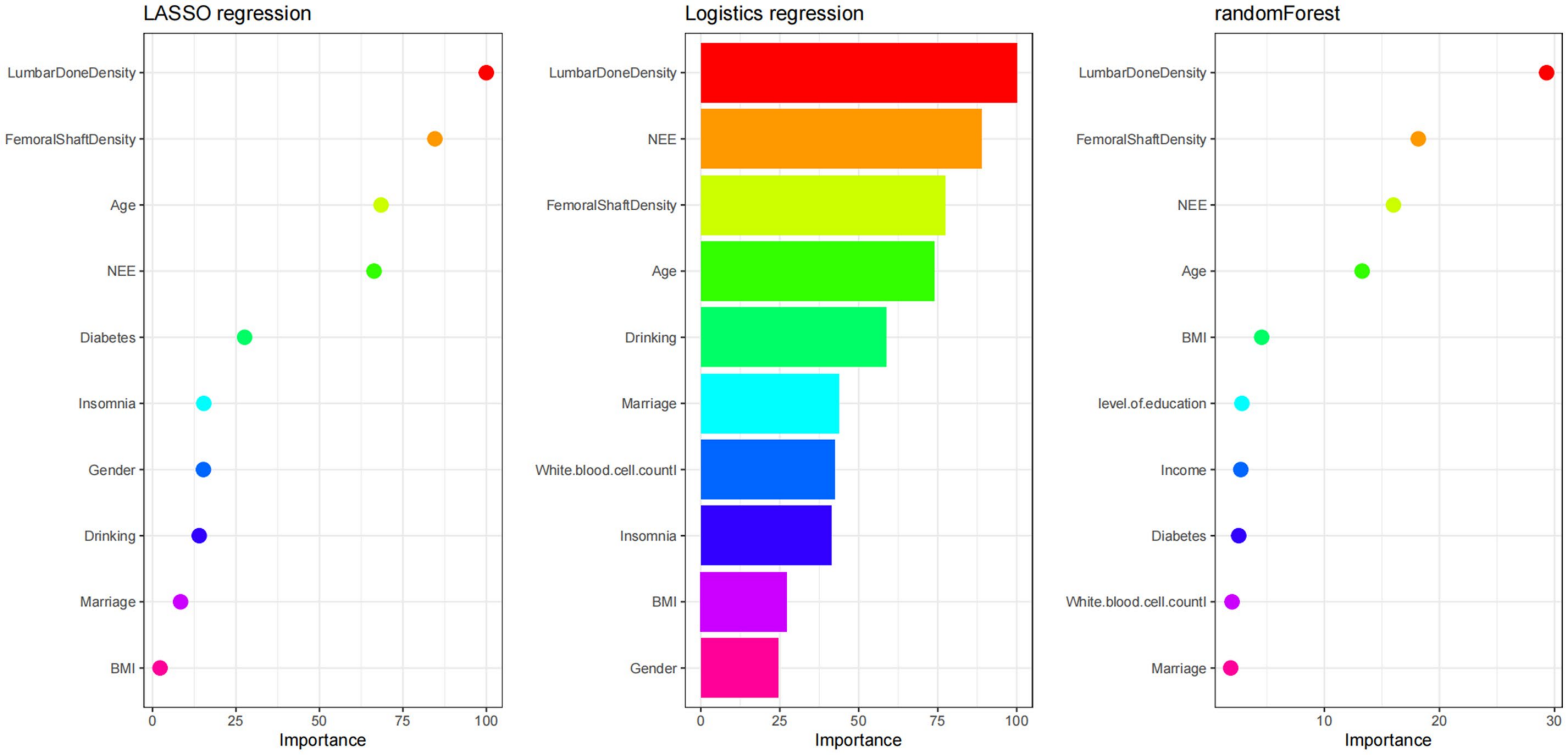
Screening for osteopenia risk factors using three feature-learning methods: LASSO regression (left), logistic regression (middle), and random forest (right).

Osteopenia was defined as a T-score between −1.0 and −2.5 standard deviations at either the lumbar spine or femoral neck. The worst-site principle was applied, whereby meeting the criterion at any site classified an individual as osteopenic. Three feature learning methods (LASSO regression, logistic regression, and random forest) were used to screen for influencing factors of osteopenia. The union of the top five influencing factors from these three methods was taken, finally resulting in seven important influencing factors. (Figure 5).

### 3.6 Analysis of the effect of osteopenia and NEE risk

Subgroup analysis revealed that the effect of NEE is prominent in the osteopenic population. Full cohort analysis showed that for each 1-unit increase in NEE, osteoporosis risk increased by 20% (OR = 1.20, 95%CI:1.16–1.44, *p* < 0.05). In the osteopenic population, the effect size of NEE was significantly higher than in the normal bone mass population (OR = 1.12 vs. 1.06), and statistical significance jumped to the *p* < 0.001 level. This between-group difference was directly supported by interaction analysis (interaction *p* = 0.0297), indicating that osteopenic status may amplify the pathological effect of nighttime eating. At a deeper mechanistic level, in the osteopenic subgroup, NEE may enhance bone metabolic sensitivity leading to abnormally elevated nocturnal bone resorption peaks, disrupting the bone resorption balance. In the subgroup, the protective effect of BMD was significantly weakened (femoral density OR = 0.10 vs. full cohort 0.33). (Table 2).

**Table 2:** Subgroup analysis of osteoporosis risk factors stratified by bone mass status: effect estimates and interaction *P* values.

Variable	Full-cohort model	Osteopenic subgroup	Normal bone mass subgroup	Interaction *p*-value
NEE	1.20 (1.6–1.44)*	1.12 (1.06–1.18)***	1.06 (1.01–11)*	0.0297
Age	1.05 (1.01–1.09)**	1.03 (1.00–1.06)**	1.07 (1.00–1.17)*	
Femoral shaft density	0.33 (0.12–0.78)**	0.10(0.01–0.19)*	0.25 (0.05–0.80)**	
Lumbar done density	0.11 (0.02–0.32)**	0.06 (0.01–5.03)**	0.17 (0.03–0.31)**	
Diabetes	1.49 (1.06–1.92)*	1.51 (0.02–3.00)*	1.33 (1.04–1.62)***	
BMI	1.01 (0.92–1.10)*	1.02 (0.96–1.8)***	1.23 (1.01–1.66)***	
Drinking	14.96 (1.72–216.83)**	17.72 (1.1–676.00)**	3.22(1.17–5.27)**	
NEE: osteopenia	−0.07942			

1. Data are presented as odds ratios (OR) with 95% confidence intervals (CI).

2. Significance markers: *P < 0.05, **p < 0.01, ***P < 0.001.

3. Interaction *p*-values are derived from the interaction term (NEE × osteopenia) in the full-cohort model.

### 3.7 Basic characteristics of NHANES research subjects

Among the 15,464 participants included, there were 7,904 males and 7,560 females, with a mean age of (38.69 ± 22.23) years and a mean NEE of (19.96% ± 9.47%). Statistically significant differences (*p* < 0.05) between groups were observed for age, race, place of birth, education level, household income, smoking history, hypertension history, diabetes history, and other data. Basic characteristic comparisons are shown in Supplementary Table 2.

### 3.8 Correlation between NEE and OP

Weighted logistic regression analysis treating NEE intake level as a continuous variable showed a correlation between NEE intake and OP (OR = 1.67, *p* = 0.002). After adjusting for variables, Model 1 did not find a correlation, while Model 2 indicated a correlation between NEE and OP. After categorizing NEE and conducting correlation analysis with OP, the crude model and Model 2 showed its correlation. The higher the NEE value, the greater the risk of developing OP. Sensitivity analysis based on the fully-adjusted Model 2, with additional adjustment for total energy intake (Model 3), showed that the NEE–OP association was only slightly attenuated but remained significant (OR = 1.79, *p* = 0.002), suggesting that the effect of NEE is independent of total energy intake (Table 3). Sensitivity analyses for other thresholds in Model 2 are presented in Supplementary Table 3.

**Table 3:** Weighted logistic regression analysis of the correlation between NEE and OP.

	Crude model		Model 1		Model 2		Model 3	
Characteristic	log(OR)95% CI	*p*-value	log(OR)95% CI	*p*-value	log(OR)95% CI	*p*-value	log(OR)95% CI	*p*-value
NEE	1.68 (1.05–2.29)	0.002	0.74 (0.423–1.063)	0.04	1.53 (1.21–1.85)	*P*<0.001	1.49 (1.18–1.80)	*P*<0.001
Q1	-	-	-	-	-	-		
Q2	1.28 (1.04–1.52)	0.012	0.76 (0.57–0.95)	0.4	1.24 (1.03–1.45)	0.012	1.28 (1.06–1.54)	0.009
Q3	2.02 (1.93–2.93)	P<0.001	0.70 (0.43–0.97)	0.3	1.83 (1.27–2.64)	0.002	1.80 (1.25–2.60)	0.003

Original model: unadjusted; Model 1: adjusted for age, gender, race, education level, and marital status; Model 2: adjusted for smoking, drinking, BMI, diabetes, hypertension, white blood cell count, NEE, femoral neck bone density, and lumbar spine bone density. Model 3: Model 2 plus total energy intake.

### 3.9 Subgroup analysis

**Figure 6:**
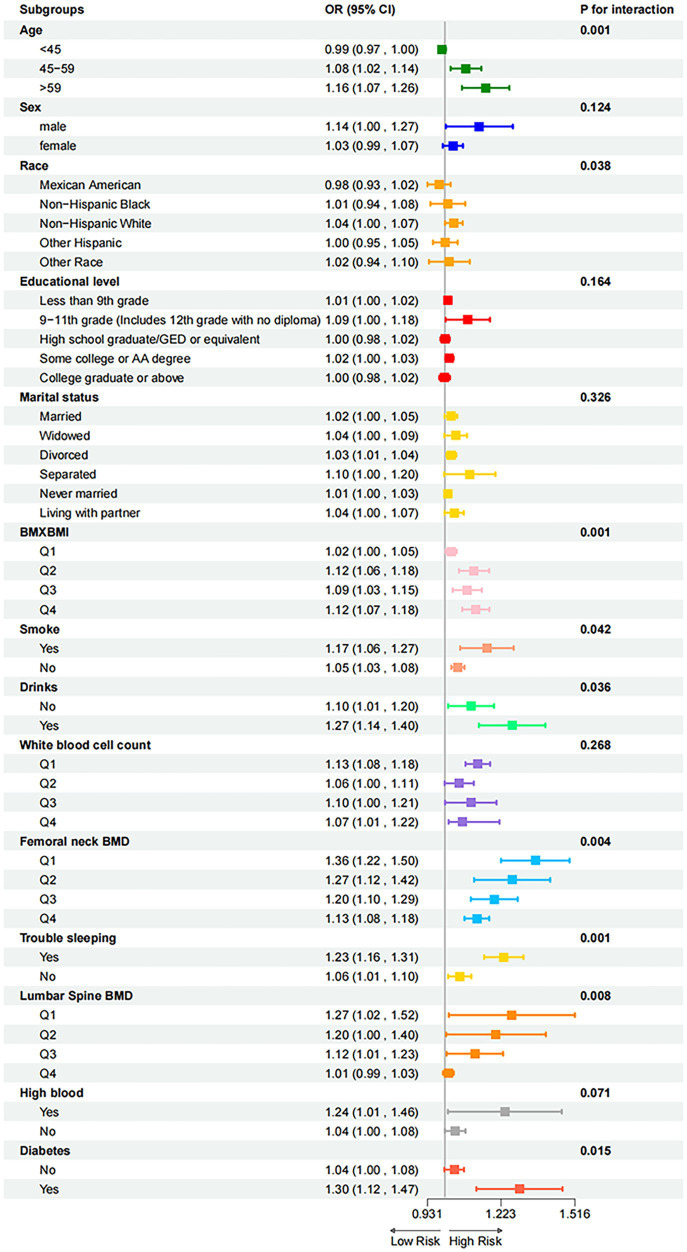
Subgroup analysis of the association between NEE and OP.

Subgroup analyses stratified by covariates were conducted using logistic regression to explore their relationships with osteoporosis (OP). Significant heterogeneity existed in the association between the exposure factor (NEE) and the outcome; the highest risk increases were observed in elderly individuals (≥60 years), those with low bone mineral density (BMD), and patients with sleep disorders (OR > 1.15). Age (*p* = 0.001), BMD (*p* ≤ 0.008), and sleep disorders (p = 0.001) were identified as significant effect modifiers (Figure 6).

### 3.10 Nonlinear plot of NEE versus OP risk

**Figure 7:**
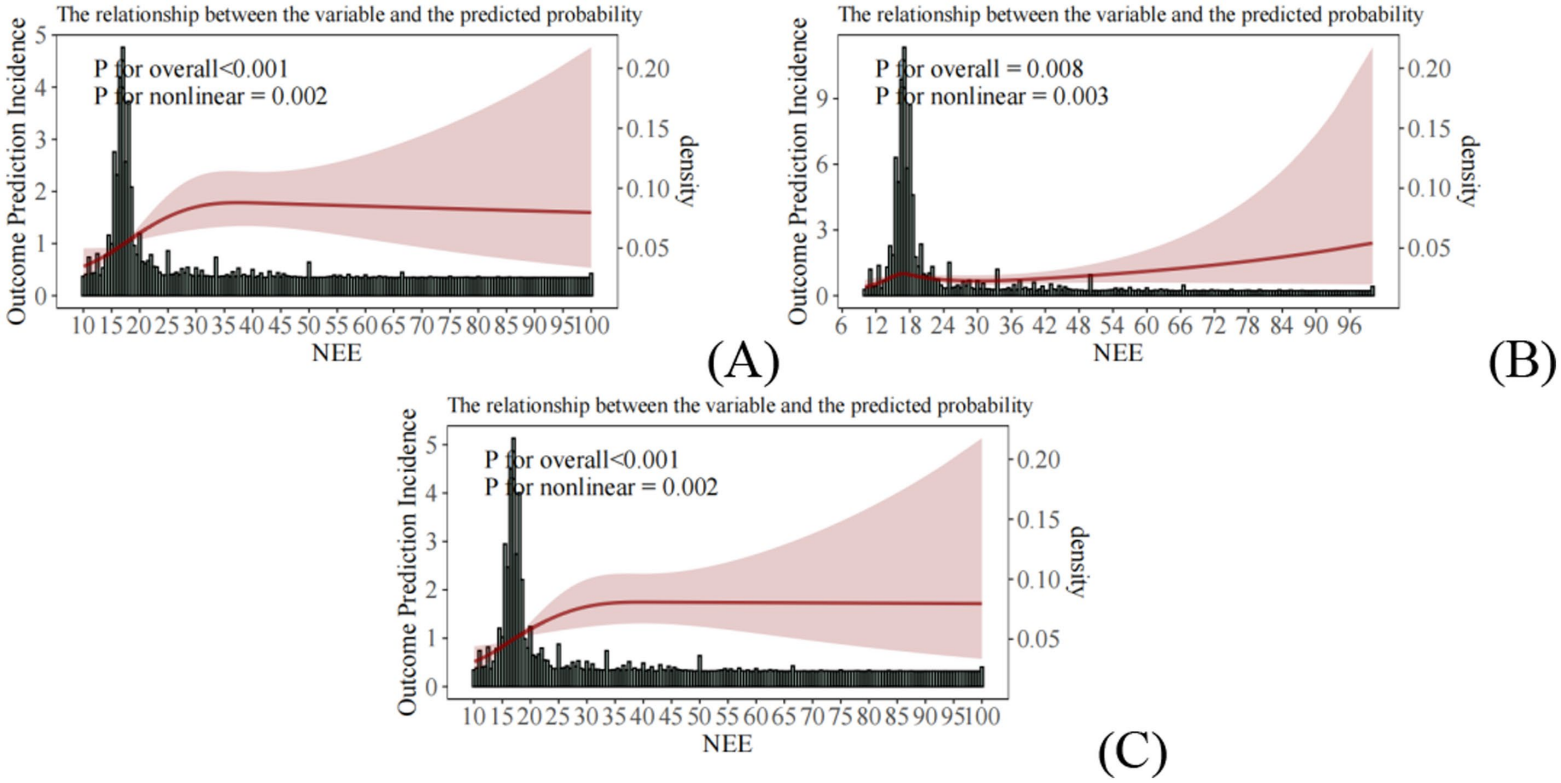
Nonlinear relationship between NEE and OP risk: **(A)** Unadjusted model; **(B)** Model 1 adjusted for age, sex, race/ethnicity, education level, and marital status; **(C)** Model 2 adjusted for smoking, alcohol consumption, BMI, diabetes, hypertension, white blood cell count, femoral neck bone mineral density, and lumbar spine bone mineral density.

A robust nonlinear association exists between NEE and OP: risk increased steeply within the NEE range of 10–25% (*p* ≤ 0.05), plateauing beyond 25%. This pattern remained highly significant (p ≤ 0.05) across the unadjusted model, demographically adjusted model, and fully adjusted model, confirming NEE as an independent predictor of bone health risk, distinct from age, sex, and metabolic factors. The 10–25% interval represents a critical threshold range for a sharp increase in risk, providing a quantitative basis for chrono-nutritional interventions (Figures 7A–C).

### 3.11 Sensitivity analysis of causality between NEE and OP

**Figure 8:**
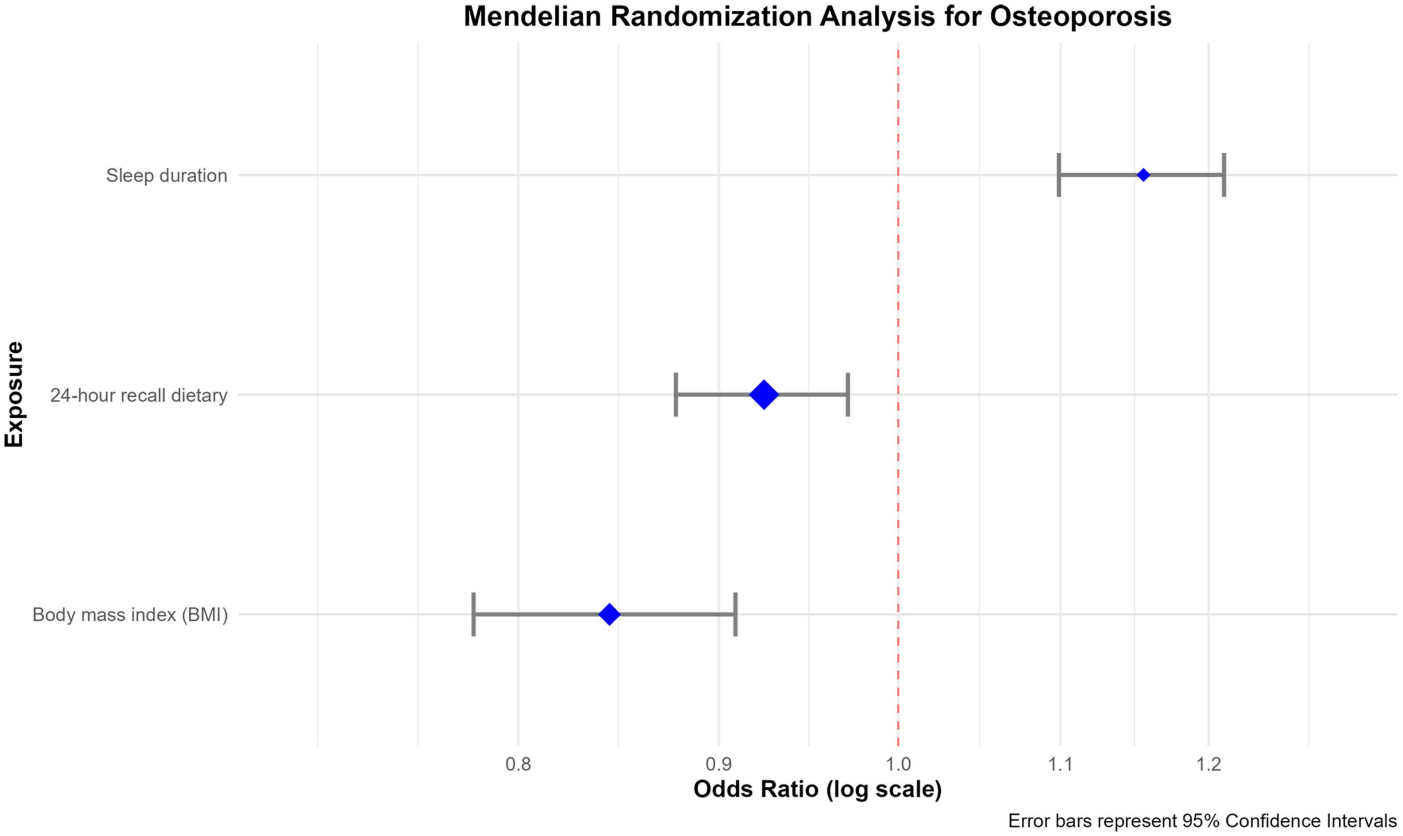
Forest plot of multivariable Mendelian randomization analysis.

Multivariable Mendelian randomization analysis showed (Figure 8): 24-h dietary pattern (*β* = 0.0228, *p* = 0.0033) and BMI were significantly positively associated with osteoporosis risk; while sleep duration was negatively associated with risk. Sensitivity analysis found no horizontal pleiotropy (MR-Egger intercept test *p* > 0.05) or significant heterogeneity (Cochran’s *Q* test p > 0.05), supporting the robustness of the results. Detailed results are in Supplementary Table 4.

## 4 Discussion

The rapid development of society has rendered shift work and nocturnal lifestyles increasingly prevalent, inevitably disrupting circadian rhythms. Existing research has established that circadian rhythm disturbances adversely affect bone metabolism, leading to reduced bone mineral density (19). Whether irregular nighttime eating exacerbates this impact warrants further investigation. By integrating clinical cross-sectional data, constructing machine learning prediction models, validating findings using the nationally representative NHANES database, and performing multivariable Mendelian randomization (MR) analysis, this study provides novel insights into the role of chrono-nutrition in skeletal health through mutually corroborative evidence from diverse methodologies.

The core finding of this study is the confirmation of NEE as an independent and quantifiable behavioral risk factor for OP. In the clinical cross-sectional study, feature selection using three machine learning methods (LASSO regression, logistic regression, and random forest) identified NEE as a significant predictor (Figure 2). While its importance was lower than that of bone mineral density (BMD, the gold standard) and age, it significantly surpassed traditional behavioral factors such as smoking and alcohol consumption. Crucially, within the constructed nomogram prediction model (Figure 3A), the combined model incorporating NEE (NEE + Femoral neck BMD + Lumbar spine BMD + Age + Alcohol consumption) demonstrated the highest predictive performance (AUC = 0.808), significantly outperforming models excluding NEE or single-variable models. This clearly indicates that NEE provides incremental predictive value beyond traditional risk factors. Validation using the NHANES database further supports this conclusion; weighted logistic regression analysis revealed that even after extensive adjustment for demographic, socioeconomic, lifestyle, metabolic disease, and BMD indicators, NEE (particularly high NEE levels, e.g., Q3 group) remained significantly positively associated with OP risk (Table 3). Nonlinear relationship analysis (Figures 7A–C) further revealed a steep increase in risk within the NEE range of 10–25%, plateauing beyond 25%, providing a preliminary quantitative threshold for defining high-risk NEE levels.

Subgroup analysis uncovered a key phenomenon: the detrimental effect of NEE on OP risk was significantly amplified in individuals with osteopenia (Table 2). Compared to those with normal bone mass, osteopenic individuals exposed to the same unit increase in NEE exhibited a greater increase in OP risk (OR = 1.12 vs. 1.06) and stronger statistical significance (*p* < 0.001 vs. *p* < 0.05). Interaction analysis (interaction *p* = 0.0297) directly confirmed an effect modification between osteopenic status and NEE. Mechanistically, this may stem from the compromised bone metabolic homeostasis in osteopenic individuals, rendering them more sensitive to the circadian disruption and metabolic dysregulation induced by NEE. This heightened sensitivity could lead to abnormally elevated nocturnal bone resorption peaks, making the delicate balance between bone formation and resorption more susceptible to disruption. Notably, within the osteopenic subgroup, the protective effect of BMD itself was significantly attenuated (femoral neck BMD OR = 0.10 vs. full cohort OR = 0.33), suggesting that NEE may partially counteract the physiological protective barrier conferred by BMD. This finding holds significant implications for precision prevention, indicating that osteopenic populations are a crucial target for NEE restriction, where early intervention may yield greater bone health benefits.

Due to the lack of genetic instrumental variables for directly studying NEE, the use of suggestive genetic evidence in MR indirectly supports the potential existence of a causal association between NEE and the risk of osteoporosis. The results (Figure 8) showed that genetic predisposition to disordered overall dietary patterns and higher BMI were both significantly positively associated with OP risk. Although not directly equivalent to the causal effect of NEE itself, this provides genetic-level causal evidence supporting the notion that “unhealthy dietary behavioral patterns (which may include NEE) and obesity are risk factors for OP,” thereby strengthening the robustness of our observational findings (20). Concurrently, longer sleep duration exhibited a protective effect, aligning with the concept that maintaining circadian health supports bone integrity. Sensitivity analyses (MR-Egger intercept test *p* > 0.05, absence of significant heterogeneity) supported the reliability of the results. Although we employed a multivariate model, it still cannot be entirely equated to the direct causal effect of NEE, providing indirect support for the existence of a potential causal relationship, but by no means conclusive evidence.

From the perspective of mechanism exploration, the association between NEE and impaired skeletal health may be realized through multiple pathways, primarily based on previous research, providing a reasonable biological explanatory framework for the findings of this study. (1) NEE may interfere with the expression of circadian rhythm clock genes: Disruption of core clock genes (such as BMAL1 and CLOCK) has been shown to inhibit osteoblast activity and potentially enhance osteoclast function, thereby disrupting the balance of bone metabolism (21). The observed association between NEE and osteoporosis (OP) in this study, particularly the heightened risk in populations with sleep disorders, indirectly supports the central role of circadian rhythm disruption in this context. (2) NEE may induce metabolic disorders: It is often accompanied by decreased insulin sensitivity, abnormal glucose tolerance, and dysfunctional adipose tissue. These metabolic alterations may indirectly and negatively regulate the bone remodeling process by upregulating inflammatory factors or influencing the secretion of key hormones in bone metabolism, such as leptin, adiponectin, and glucocorticoids (22). The significant association observed in this study, even after adjusting for metabolic-related factors such as diabetes and hypertension, suggests that NEE may impact bone health through independent or overlapping metabolic pathways. (3) It is speculated that NEE may inhibit melatonin secretion: Nocturnal eating is often associated with nighttime light exposure, which can suppress melatonin secretion, a hormone known for its direct bone-protective effects (23, 24). Its deficiency may further impair bone mineralization capacity. Although this study did not directly measure melatonin levels, the temporal characteristics of NEE (from 8 p.m. to 6 a.m.) suggest it could be an important mechanistic link.

Therefore, we speculate that the harm of NEE to bone is the result of the combined action of the “timing factor” and the “possibly accompanying food quality factor.”

### 4.1 Advantages and limitations

The main strength of this study lies in its comprehensive research strategy: it integrates nationally representative observational data from NHANES, Mendelian randomization analysis for causal inference, and the construction and validation of diagnostic models in independent clinical cohorts, providing multi-level evidence. In the observational analysis, a wide range of covariates, including key bone mineral density indicators, were adjusted. It reveals nonlinear relationships and effect modifications: it clarifies the nonlinear dose–response relationship between NEE and OP and identifies high-risk populations (such as those with low bone mineral density, sleep disorders, etc.). It has certain clinical translatability, as it constructs and validates a practical clinical diagnostic prediction model for NEE.

Limitations include: The cross-sectional design of the clinical component precludes establishing the absolute temporal sequence and long-term effects of NEE on OP. Although Mendelian randomization (MR) was used to infer causality, the indirect exposure variables cannot fully equate to the direct causal effect of NEE. The definition and measurement of NEE (8:00 p.m. to 6:00 a.m.) were based on literature review and habits, lacking an internationally unified standard. Reliance on 24-h dietary recall introduces potential recall bias and measurement error. Specific food types, quality, and special circumstances like shift workers were not differentiated. The clinical cross-sectional study had a relatively small sample size (*n* = 186) from a single center (China), necessitating caution in extrapolation. While NHANES provided a large, nationally representative sample, its US population has cultural and dietary differences. Despite adjusting for multiple confounders in statistical models, residual confounding from unknown or unmeasured factors (e.g., detailed physical activity levels, specific nutrient intakes, other sleep parameters) may influence the results.

## 5 Conclusion

In summary, supported by multiple evidence chains, this study confirms that NEE is an independent and potentially modifiable risk factor for OP, with particularly significant harm for the osteopenic population. NEE may impair skeletal health through multiple pathways, including disrupting circadian rhythms, inducing metabolic dysregulation, and affecting the secretion of key hormones (e.g., melatonin). The research results provide a new intervention direction for incorporating “dietary timing management” into comprehensive osteoporosis prevention and control strategies, especially for precision intervention targeting high-risk osteopenic populations. Future research should focus on elucidating its deeper mechanisms and validating the effectiveness of adjusting eating chronology for improving skeletal health in different populations.

## Data Availability

The original contributions presented in the study are included in the article/Supplementary material, further inquiries can be directed to the corresponding author.
