# Supplementary material for: Development and validation of an osteoporosis risk prediction model incorporating nighttime eating exposure
Source: Front Nutr. 2025 Nov 21;12:1660080. doi: 10.3389/fnut.2025.1660080 (PMC12678082; doi:10.3389/fnut.2025.1660080)
Supplement: Supplementary file 1 [file Data_Sheet_1.docx]

Supplementary Material

**Supplementary Table 1. Bootstrap validation results of the nomogram prediction model (1,000 resamples)**

| **Performance metrics** | **Raw value** | **Bootstrap-corrected value** | **Optimism** |
| --- | --- | --- | --- |
| C-index | 0.808 | 0.793 (95% CI: 0.768-0.818) | 0.015 |

**Supplementary Table 2 Demographic Baseline Chart**

| **Characteristic** | **Overall**  N = 253,998,627^1^ | **Q1** N= 86,780,155^1^ | **Q2**  N = 80,863,483^1^ | **Q3**  N = 86,354,988^1^ | **p-value**^3^ |
| --- | --- | --- | --- | --- | --- |
| **Age** | 38.69± (22.23) | 38.89± (25.00) | 42.87± (20.17) | 34.57± (20.26) | <0.001 |
| **Gender** |  |  |  |  | <0.001 |
| Male | 7,904 (51%) | 2,250 (41%) | 2,340 (57%) | 3,314 (55%) |  |
| Female | 7,560 (49%) | 2,964 (59%) | 1,838 (43%) | 2,758 (45%) |  |
| **Ethnicity** |  |  |  |  | <0.001 |
| Mexican American | 2,433 (11%) | 830 (10%) | 624 (10%) | 979 (12%) |  |
| Other Hispanic | 1,407 (6.5%) | 546 (7.7%) | 365 (5.9%) | 496 (5.9%) |  |
| Non-Hispanic White | 5,433 (61%) | 2,099 (64%) | 1,547 (65%) | 1,787 (55%) |  |
| Non-Hispanic Black | 3,429 (12%) | 692 (7.2%) | 918 (11%) | 1,819 (17%) |  |
| Other Race Including Multi-Racial | 2,762 (9.9%) | 1,047 (11%) | 724 (8.8%) | 991 (9.9%) |  |
| **Level of education** |  |  |  |  | <0.001 |
| Less Than 9th Grade | 980 (3.6%) | 448 (4.5%) | 306 (4.0%) | 226 (2.5%) |  |
| 9-11th Grade (Includes 12th grade with no diploma) | 1,616 (8.0%) | 432 (6.9%) | 438 (7.8%) | 746 (9.2%) |  |
| High School Grad/GED or Equivalent | 3,940 (25%) | 964 (20%) | 931 (22%) | 2,045 (32%) |  |
| Some College or AA degree | 5,724 (36%) | 1,875 (34%) | 1,544 (36%) | 2,305 (37%) |  |
| College Graduate or above | 3,190 (28%) | 1,489 (34%) | 956 (30%) | 745 (19%) |  |
| **Marriage** |  |  |  |  | <0.001 |
| Married | 6,663 (48%) | 3,122 (59%) | 2,041 (53%) | 1,500 (33%) |  |
| Widowed | 687 (4.2%) | 276 (6.0%) | 244 (4.0%) | 167 (2.5%) |  |
| Divorced | 1,067 (8.1%) | 356 (8.2%) | 326 (8.6%) | 385 (7.4%) |  |
| Separated | 283 (1.7%) | 101 (1.7%) | 83 (1.6%) | 99 (1.9%) |  |
| Never married | 5,978 (31%) | 1,186 (20%) | 1,210 (26%) | 3,582 (48%) |  |
| Living with partner | 786 (6.2%) | 173(4.1%) | 274 (7.2%) | 339 (7.3%) |  |
| **BMI** | 27.20± (7.79) | 25.55± (7.06) | 28.34± (7.17) | 27.79± (8.72) | <0.001 |
| **Household income** | 2.87± (1.63) | 2.99± (1.58) | 3.07± (1.63) | 2.55± (1.63) | <0.001 |
| **Smoking** |  |  |  |  | <0.001 |
| NO | 4,101 (33%) | 1,056 (26%) | 1,425 (38%) | 1,620 (36%) |  |
| YES | 11,363 (67%) | 4,158 (74%) | 2,753 (62%) | 4,452 (64%) |  |
| **Drinking** |  |  |  |  | <0.001 |
| YES | 13256(85.7%) | 3314(21.4%) | 6628(42.7%) | 9942(64.3%) |  |
| NO | 2205(14.3%) | 552(3.6%) | 1103(7.1%) | 1505(9.7%) |  |
| **White blood cell count** | 7.42± (3.01) | 7.38± (4.10) | 7.35± (2.10) | 7.53± (2.35) | 0.033 |
| **Femoral neck BMD** | 0.78± (0.11) | 0.74± (0.10) | 0.80± (0.11) | 0.81± (0.12) | <0.001 |
| \| **Lumbar Spine BMD** \| \| --- \| | 0.97± (0.17) | 0.92± (0.16) | 1.00± (0.14) | 1.00± (0.19) | <0.001 |
| **NEE** | 19.96± (9.47) | 15.34± (1.40) | 17.18± (0.38) | 27.22± (13.43) | <0.001 |
| **Hypertension** |  |  |  |  | <0.001 |
| YES | 3,468 (26%) | 1,087 (25%) | 1,220 (30%) | 1,161 (22%) |  |
| NO | 11,996 (74%) | 4,127 (75%) | 2,958 (70%) | 4,911 (78%) |  |
| **Diabetes** |  |  |  |  | <0.001 |
| YES | 1,273 (8.1%) | 386 (7.7%) | 443 (9.4%) | 444 (7.2%) |  |
| NO | 13,882 (90%) | 4,779 (91%) | 3,615 (88%) | 5,488 (90%) |  |
| Borderline | 309 (2.1%) | 49 (0.9%) | 120 (2.9%) | 140 (2.5%) |  |
| **Osteoporosis** |  |  |  |  |  |
| YES | 584 (4.3%) | 244 (5.6%) | 179 (4.5%) | 161 (2.9%) |  |
| NO | 14,880 (96%) | 4,970 (94%) | 3,999 (95%) | 5,911 (97%) |  |
| ^1^Mean± (SD); n (unweighted) (%) | | | | | |
| ^2^Design-based KruskalWallis test; Pearson's X^2: Rao & Scott adjustment | | | | | |

# Note: Participants were divided into Q1 (low), Q2 (medium), and Q3 (high) groups based on the tertiles of NEE percentage.

# **Supplementary Table 3 OP risk in the highest NEE exposure group at different NEE cut-offs (fully-adjusted model)**

| NEE | log(OR)95% CI | p-value |
| --- | --- | --- |
| >5% | 1.42 (1.08-1.86) | 0.012 |
| >10% | 1.58 (1.19-2.10) | 0.002 |
| >15% | 1.69 (1.25-2.28) | 0.001 |
| >20% | 1.76 (1.28-2.42) | 0.001 |
| >25% | 1.83 (1.27-2.64) | 0.002 |

Note: All models were adjusted for age, sex, race, education, marital status, smoking, alcohol use, BMI, diabetes, hypertension, white blood cell count, femoral neck BMD, and lumbar spine BMD. Sample size represents the weighted estimate from the NHANES 2013–2014 and 2017–2018 cycles.

**Supplementary Table 4. Sensitivity statistics for multivariable Mendelian randomization analysis**

| Exposure variable | F | Cochran's Q P-value (IVW) | I² (%) | MR-Egger intercept | P-value (Egger intercept) |
| --- | --- | --- | --- | --- | --- |
| 24-hour dietary pattern | 31.2 | 0.18 | 19.5 | -0.006 | 0.42 |
| BMI | 42.7 | 0.11 | 16.8 | 0.004 | 0.38 |
| Sleep duration | 28.3 | 0.24 | 12.3 | 0.002 | 0.51 |

**Supplementary Figure 1.**


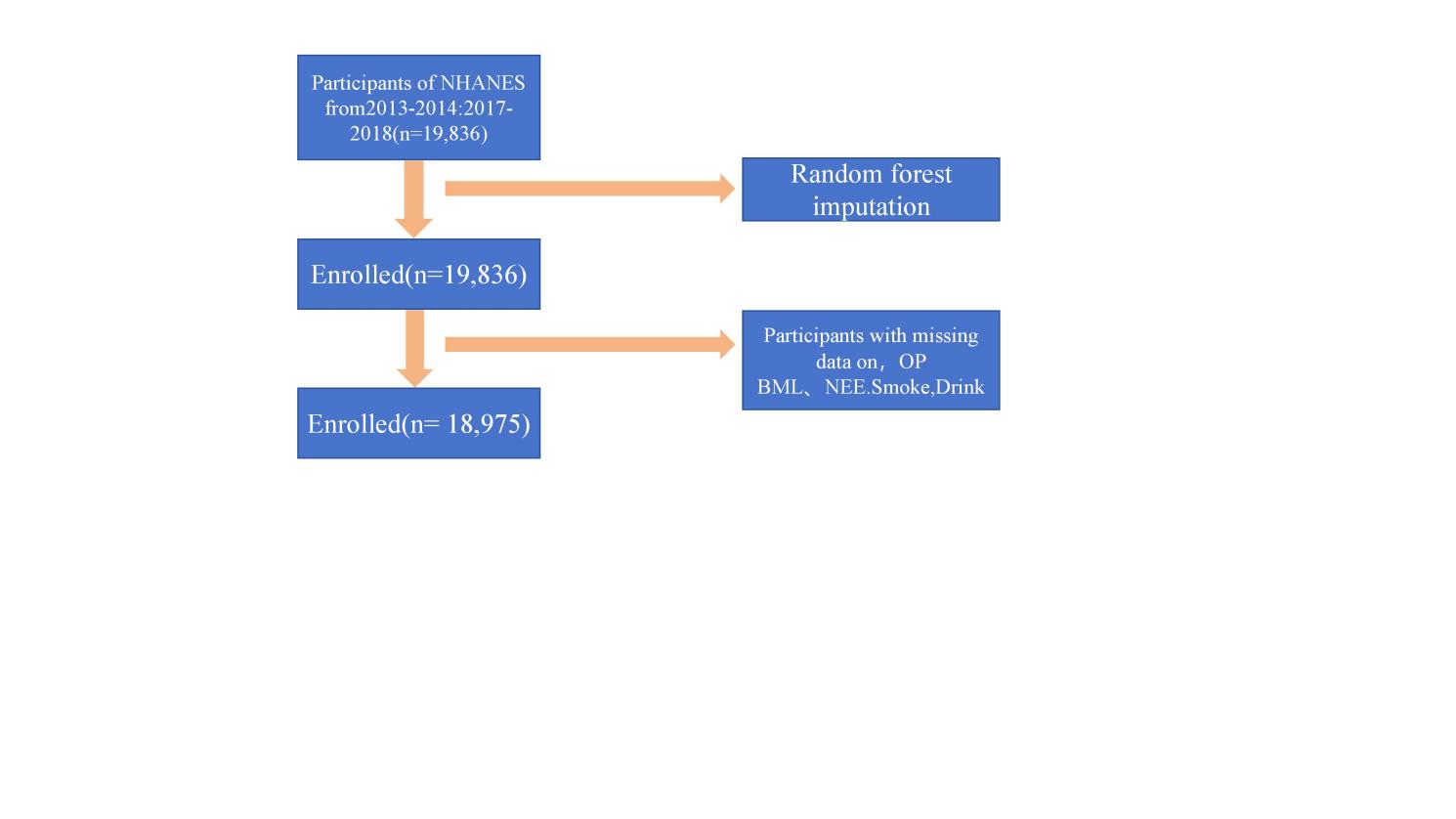


Supplementary Figure 1. Flowchart illustrating the stepwise participant selection process from the 2013–2014 and 2017–2018 cycles of the National Health and Nutrition Examination Survey (NHANES）
